# Evolution of parental care in haploid–diploid plants

**DOI:** 10.1098/rspb.2023.2351

**Published:** 2024-02-14

**Authors:** Kazuhiro Bessho, Akira Sasaki

**Affiliations:** ^1^ Medical Research Center, Saitama Medical University, 38 Morohongo Moroyama-machi, Iruma-gun, Saitama 350-0495, Japan; ^2^ Research Center for Integrative Evolutionary Science, The Graduate University for Advanced Studies, SOKENDAI, Hayama, Kanagawa 240-0193, Japan; ^3^ Evolution and Ecology Program, International Institute for Applied Systems Analysis, Schlosplatz 1, Laxenburg 2361, Austria

**Keywords:** haploid–diploid life cycle, alternation of generations, parental care, genomic conflict, bryophyte, carposporophyte

## Abstract

In bryophytes that alternate between haploid gametophytes and diploid sporophytes through sexual reproduction, sporophytes are often attached to and nurtured on the female gametophyte. A similar phenomenon is seen in Florideophyceae (a group of red algae). These systems in which a gametophyte (mother) invests nutrients in sporophytes (offspring) are ideal for studying the evolution of ‘parental care’ in non-animal organisms. Here, we propose a model of a haploid–diploid life cycle and examine the evolution of maternal investment in sporophytes focusing on two effects: the degree of paternal or maternal control of investment and the number of sporophytes. We find that when the female dominantly controls the investment, the evolutionarily stable level of investment is that which maximizes the expected reproductive success of the female gametophyte. The genomic conflict between maternal and paternal alleles complicates the evolutionary outcome; however, a greater male allelic effect and a higher number of sporophytes favour a higher energy investment, which may lead to evolutionary branching or run-away escalation of the investment level. This suggests that the selfishness of the paternal gene is the evolutionary driver of parental care and that complex structures such as fusion cells in red algae may have evolved to suppress it.

## 1. Introduction

The evolution of parental care is one of the most investigated topics in behavioural ecology. Because this phenomenon significantly involves interactions between individuals (e.g. male and female, parent and child), evolutionary game theory has guided our understanding of the evolution of parental care in animals [1–5].

Compared to our understanding of parental care in animals, less is known about the evolution of parental care in plants. In recent years, however, the application of behavioural ecological concepts to plants has received increasing attention. A particularly important related subject is the genomic conflicts and the evolution of double fertilization and endospermy in seed plants. In seeds produced by seed plants, the embryo is surrounded by a special tissue called the endosperm that develops from an independent fertilization event (double fertilization). It is known that in an endosperm, which supplies energy to the embryo, the maternal genome is often duplicated. This interesting phenomenon has been explained from the viewpoint of genomic conflict [6,7]; it has been hypothesized that polyploidy of the maternal genome is favoured to prevent the selfishness of the paternal genomic effect from excessively increasing the maternal investment.

In this article, we focus on a phenomenon that can be described as more direct parental care in plants. Eukaryotic organisms exhibit diverse life cycles associated with their changes in ploidy between stages. The most common life cycle in macroalgae and terrestrial plants is a biphasic haploid–diploid life cycle [8,9]. In this life cycle, a haploid multicellular stage (gametophyte) alternates with a diploid multicellular stage (sporophyte). In many macroalgae, gametophytes and sporophytes grow independently (free-living), but in some groups, the diploid sporophytes often grow while attached to the haploid gametophyte [10].

**Figure 1.**
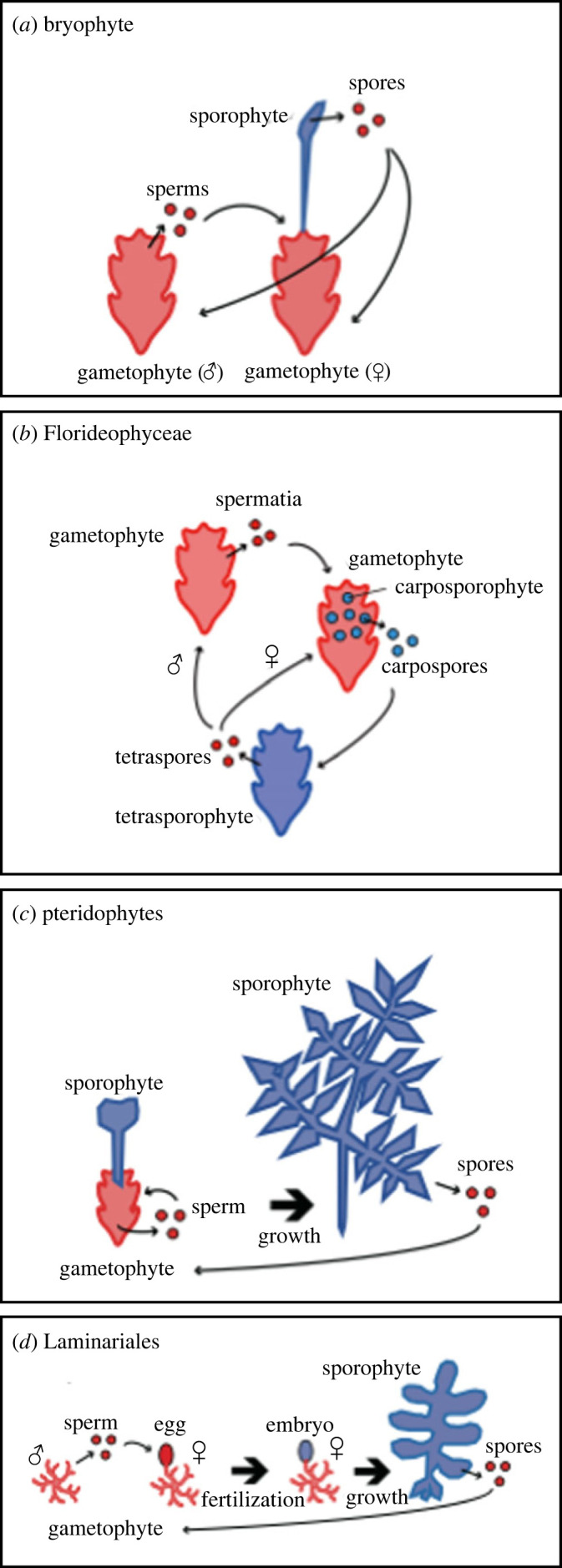
Diagram of typical life cycles of (*a*) bryophyte, (*b*) Florideophyceae, (*c*) pteridophytes and (*d*) Laminariales. Red indicates haploids and blue indicates diploids.

For example, bryophytes typically have a haploid-dominant haploid–diploid life cycle. In this life cycle, swimming sperm, the dispersal of which is helped by microarthropods [11,12], fertilize the eggs on female gametophytes and the fertilized eggs develop into diploid sporophytes on the maternal haploid bodies (figure 1*a*). The attached diploid sporophyte is nutritionally dependent on its haploid mother [10].

A similar phenomenon can be found in a different lineage, red algae (rhodophytes). One of the dominant groups of red algae, Florideophyceae, tends to exhibits a triphasic life cycle [13–16]. In this life cycle, generations alternate among three distinguishable stages: haploid gametophyte, diploid carposporophyte and diploid tetrasporophyte. Syngamy occurs on the parent female gametophyte when it randomly encounters a sperm from the male gametophyte. The fertilized zygotes develop into carposporophytes, the first diploid stage. A carposporophyte then produces diploid carpospores by mitosis and carpospores develop into tetrasporophytes, the second free-living diploid stage. Finally, haploid tetraspores produced from a tetrasporophyte through meiosis develop into gametophytes (figure 1*b*). We note that species in other groups (e.g. Bangiophyceae) exhibit the biphasic life cycle with free-living stages and they do not present parental care.

We further note that similar phenomena may occur in pteridophytes and brown algae (phaeophyceae). Unlike bryophytes, pteridophytes exhibit a diploid-dominant haploid–diploid life cycle. In some species (e.g. fern *Osmunda claytonia*), the sporophyte attaches to the gametophyte during early development, and when the gametophyte dies, the sporophyte begins to grow independently (figure 1*c*) [10,17]. Similarly, species in one of the groups of brown algae, Laminariales, exhibits a diploid-dominant cycle. In sexual reproduction, an egg attached to the female gametophyte is fertilized, from which the sporophyte develops (figure 1*d*). Their diploid sporophytes are long-lived and ultimately achieve nutritional independence, although early in development they would be briefly dependent upon a gametophyte.

In these life cycles, the diploid sporophyte that is attached to the haploid gametophyte is often nurtured and sheltered by the maternal gametophyte [10,13,18]. Classically, this ‘parental care’ in algae and plants is understood by the hypothesis of Searles [13], who proposed that the triphasic life cycle in red algae evolved as a way to compensate ecologically for a low fertilization success rate caused by the lack of motile gametes. Red algae never form flagella, and fertilization by spermatia (immotile sperm) depends strongly on unpredictable water flows; thus, the decreased probability of fertilization favours greater resource allocation to the rare, successfully fertilized zygotes. Searles [13] also suggested that parental care has also evolved in green plants (bryophytes and pteridophytes) in response to similar evolutionary forces. Because fertilization by swimming sperm would occur more rarely in a terrestrial environment than in an aquatic environment, evolution would favour parental care to compensate for the low fertilization rate [13].

However, empirical studies [19–21] that worked on red algae estimated very high fertilization success, and questioned the Searles hypothesis. Furthermore, the presence of pollinators has recently been discovered in red algae [22]. We, therefore, consider another hypothesis about the evolution of parental care. While Searles’ hypothesis implicitly assumes that the maternal gametophyte fully controls the level of ‘paternal care’, this interesting phenomenon of maternal nutrient supply set the stage in the cared diploid sporophytes for genomic conflict between maternal and paternal genes as in the evolution of duplicate fertilization [10,23,24]. The diploid sporophyte inherits both maternal and paternal alleles from its parents. In dioicous species female gametophytes supply resources to the sporophytes but male gametophytes do not, leading to sexual conflict in the diploid sporophytes and possibly to the evolution of genomic imprinting [10]. Here, we aim to formulate these verbally expressed theories into a mathematical model to clarify the evolutionary forces that drive the evolution of parental care in red algae, bryophytes (mosses), and pteridophytes (ferns). By analysing our mathematical model, we examine the conditions under which evolutionary forces favour parental care in haploid–diploid plants. Specifically, we test the hypothesis that genomic conflict within diploid sporophytes has led to the evolution of parental care.

## 2. Model

**Figure 2.**
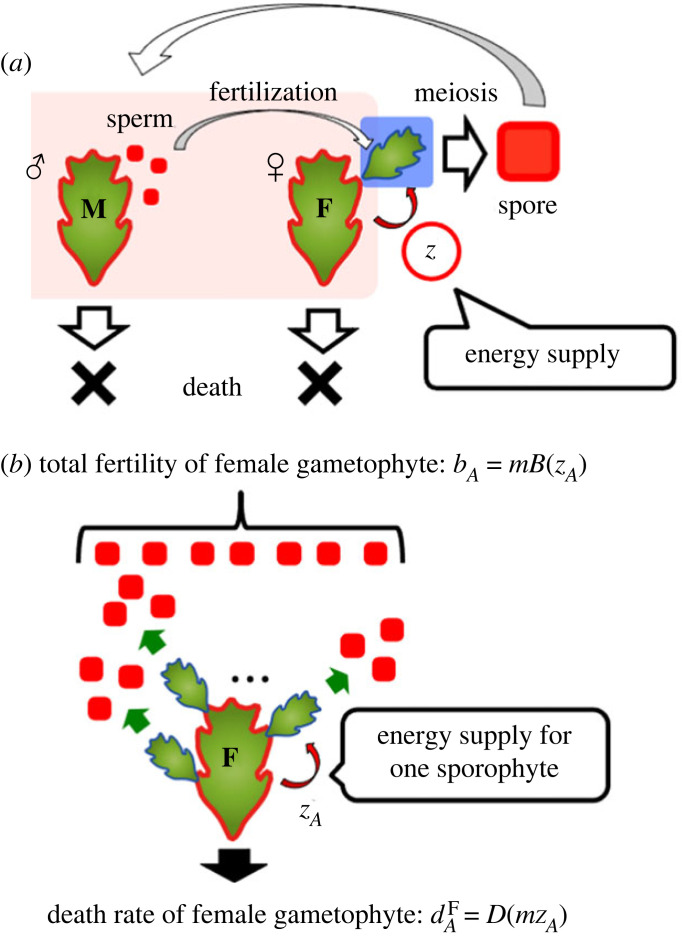
Schematic illustration of (*a*) the bryophyte life cycle and (*b*) reproduction and mortality of female gametophytes. The population consists of male (M) and female (F) gametophytes. By contrast to the independent haploid gametophytes, the diploid sporophyte physically and nutritionally develops on the maternal body. The female gametophyte invests energy amount *z* in the sporophytes, and the sporophytes produce haploid spores by meiosis. We assume half of the spores develop into female gametophytes. When the resident allele *A* is fixed in the population, the energy investment of a female gametophyte is *z_A_*, and the total amount of parental care invested by a female gametophyte is *mz_A_*. As a result, the total reproduction rate of a female gametophyte is *b_A_* = *mB*(*z_A_*) and the mortality rate is $d_{A}^{F}=D(mz_{A})$.

To understand the evolution of parental care in haploid–diploid plants in terms of the hypotheses by Searles [13] and Haig & Wilczek [10], we developed a population genetic model that considers the ecological dynamics of species with a haploid–diploid life cycle. For tractability, we assumed that traits in the modelled species are controlled by a one-locus system. Let the modifier gene that decides the amount of resources invested by a female gametophyte in the offspring (sporophytes) on its body have a resident allele *A* and a mutant allele *a*. We examine the evolution of parental care in a species with a haploid–diploid biphasic life cycle (dioicous bryophyte life cycles; figure 2*a*) in the main body, but we obtain similar results for species with a triphasic life cycle (see electronic supplementary material, Mathematica file S1, for the derivation of the condition for the invasibility of a mutant in species with a triphasic life cycle).

### (a) Resident dynamics

We first consider the case where the resident allele *A* is fixed in the population. In bryophytes that have separate sexes at the gametophyte stage, a sporophyte will make two male and two female spores by meiosis. Hence, we assume that both sexes get equal investment. For mathematical tractability, we assume that sexual reproduction is obligate, the amount of sperm is not limited (so that all female gametophytes can find mates), and the sporophyte stage fully depends on the female gametophyte for its nutrition.

The population dynamics for a female gametophyte *F_A_*(*t*) and male gametophyte *M_A_*(*t*) can be described as

2.1a $$\frac{dF_{A}}{dt}=\gamma (t)\left(\frac{b_{A}}{2}F_{A}(t)\right)-d_{A}^{F}F_{A}(t)$$

and

2.1b $$\frac{dM_{A}}{dt}=\gamma (t)\left(\frac{b_{A}}{2}F_{A}(t)\right)-d^{M}M_{A}(t),$$

where *b_A_* is the fertility of a female gametophyte with allele *A* that invests energy *z_A_* in its offspring, and $d_{A}^{F}$ and *d*^M^ are the mortalities of the female and male gametophytes. The female's fertility *b_A_* and mortality $d_{A}^{F}$ depend on the energy supply level (*z_A_*) of genotype (*A*) from the female gametophyte to its offspring (sporophytes), as described later. The factor 1/2 in front of *b_A_* is due to the assumption of an equal sex ratio. The normalization factor $\gamma (t)=(d_{A}^{F}F_{A}(t)+d^{M}M_{A}(t))/b_{A}F_{A}(t)$ represents a strict density dependence such that total production of newborns is equal to total deaths in the population. As a result of this normalization, the total frequency of female and male gametophytes is always 1: *F_A_*(*t*) + *M_A_*(*t*) = 1.

When we consider the population dynamics of residents, the male has the same allele *A* as the female; hence, we do not need to consider the effect of genomic conflict on investment *z*. Therefore, we can describe the fertility of female gametophytes as

2.2 $$\begin{array}{c} b_{A}=mB(z_{A}), \end{array}$$

where *m* indicates the number of sporophytes (fertilized eggs) on a female gametophyte, and the function *B*(*x*) indicates the number of cells that a sporophyte produces via meiosis (or mitosis) that receive energy *x* from the parent gametophyte. We assume that d*B*/d*x* > 0: that is, the greater the investment in parental care, the greater the fertility of the female gametophyte. We can also describe the mortality of the female gametophyte under the haploid (maternal) determination as

2.3 $$\begin{array}{c} d_{A}^{F}=D(mz_{A}), \end{array}$$

where *mz_A_* indicates the total amount of parental care invested by a female gametophyte with genotype *A* in its offspring. We assume that female gametophytes have enough energy for this investment so that the residual energy never becomes negative. We also assume that d*D*/d*x* > 0: that is, the greater the amount of parental care invested, the greater the mortality of the female gametophyte. The evolution of nutrient investment is thus strongly constrained by a trade-off because increasing the investment in the sporophyte increases both the fertility and mortality of the female gametophyte (figure 2*b*).

In our numerical examples, we assume the following specific forms by which the fertility *B*(*x*) of a sporophyte and the mortality *D*(*x*) of the female gametophyte depend on the energy supply level *x*:

2.4 $$\begin{array}{c} B(x)=c_{B}x^{\beta }\;\;\mathrm{and}\;\;D(x)=d_{0}+c_{D}x, \end{array}$$

where *c*_B_, *β*, *d*_0_, and *c*_D_ are constant positive parameters; the fertility of a sporophyte *B*(*x*) increases with the energy *x* invested in it but with diminishing return (*β* < 1), whereas the mortality of the female gametophyte increases linearly with the total amount of energy it invests in its offspring.

When the resident population reaches an equilibrium of dynamics (2.1), the frequencies of the female and male gametophytes should be

2.5 $$\begin{array}{c} {\hat{F}}_{A}=\frac{d^{M}}{d^{M}+d_{A}^{F}}\;\;\;\;\;\mathrm{and}\;\;\;\;{\hat{M}}_{A}=\frac{d_{A}^{F}}{d^{M}+d_{A}^{F}}. \end{array}$$

### (b) Invasion analysis

Using the equilibrium state expressed by (2.5), we can derive the condition under which the resident allele *A* refuses the invasion of a mutant allele *a*. When we consider the mating scheme, we assume that there are *m* ‘slots’ (archegonia or carpogonia) on each female gametophyte that can be mated. To simplify the mating process, we assume that the slots in a female gametophyte are rapidly fertilized by sperms. Furthermore, once a slot is mated, the female sporophyte continues to produce spores of the same genotype from the slot by clonally amplifying the first fertilized egg.

If we consider the dynamics of a rare mutant allele *a*, there are three possibilities regarding its state in the mutant population: it may occur as a mutant female gametophyte (*F_a_*), as a mutant male gametophyte (*M_a_*), or as one of *m* sporophytes with genotype *Aa* on a resident female gametophyte $\left({\tilde{F}}_{A}\right)$. Because the mutant allele is assumed to be rare, we can ignore the possibility that a resident female gametophyte has two or more *Aa* sporophytes, or that a mutant female gametophyte has an *Aa* sporophyte. We can thus describe the dynamics of a rare mutant as (2.6) (see electronic supplementary material, appendix A, for the detailed derivation):

2.6a $$\begin{array}{c} \frac{d}{dt}\left(\begin{array}{c} {\tilde{F}}_{A}\\ F_{a}\\ M_{a} \end{array}\right)=\left(\begin{array}{ccc} -\tilde{D} & 0 & g\\ p & q-D(mz_{A|a}) & 0\\ p & q & -d^{M} \end{array}\right)\left(\begin{array}{c} {\tilde{F}}_{A}\\ F_{a}\\ M_{a} \end{array}\right), \end{array}$$

where $\tilde{D}=D((m-1)z_{A}+z_{a|A})$ and

2.6b $$p=\frac{\hat{\gamma }}{4}B(z_{a|A})=\frac{1}{2m}\frac{D(mz_{A})}{B(z_{A})}B(z_{a|A}),$$

2.6c $$q=\frac{\hat{\gamma }m}{4}B(z_{A|a})=\frac{1}{2}\frac{D(mz_{A})}{B(z_{A})}B(z_{A|a})$$

2.6d $$\mathrm{and}\;g=\frac{\hat{\gamma }m}{2}\frac{{\hat{F}}_{A}}{{\hat{M}}_{A}}mB(z_{A})=md^{M}.$$

Here we used $\hat{\gamma }=(2D(mz_{A}))/(mB(z_{A}))$ and ${\hat{F}}_{A}/{\hat{M}}_{A}=$ $d^{M}/d_{A}^{F}=d^{M}/D(mz_{A})$ that hold at the equilibrium population of the resident. We call the matrix in (2.6*a*) the ‘invasion matrix’ for the mutant allele. Owing to the genomic conflict between maternal and paternal allelic effects, the energy supply level to the sporophyte with genotype *Aa* depends on the sex of the gametophyte that provided the mutant allele. Here, we denote the origin of the allele by its position relative to a vertical bar ‘|’: the allele from the male is shown on the left side of the bar, and the allele from the female is shown on the right. For example, when the mutant allele *a* is inherited from the male gametophyte and the resident allele *A* is inherited from the female gametophyte, the energy supply level from the female gametophyte to the *Aa* sporophyte is described as *z_a_*_|_*_A_*, whereas if the mutant allele *a* is inherited from the female gametophyte and the resident allele *A* is inherited from the male gametophyte, the energy supply level is described as *z_A_*_|_*_a_*. Solving the characteristic equation of the invasion matrix (the 3 × 3 matrix on the right-hand side of (2.6*a*)), we obtain the condition under which the resident allele *A* refuses the invasion of the mutant allele *a* when the mutant trait is similar to the resident trait (see Results).

### (c) Evolutionary dynamics under genomic conflict

We examine the evolutionary dynamics of a quantitative trait 0 ≤ *z* ≤ *z*_max_ (energy supply level) under a genomic conflict, where *z*_max_ indicates the biological upper limit of the energy supply that is decided by the availability of resources to the maternal gametophyte. When we analyse the model, we assume that the amount of energy supplied by the gametophyte to the sporophyte under a genomic conflict is realized as the weighted mean of the paternal and maternal allelic effects, *z_X_* and *z_Y_*, where *X* and *Y* are allelic states of paternal and maternal genomes:

2.7 $$\begin{array}{c} z_{X|Y}=kz_{X}+(1-k)z_{Y}, \end{array}$$

where *k* indicates the weight of the paternal allelic effect and 1 − *k* that of the maternal allelic effect (0 ≤ *k* ≤ 1). Thus, when the maternal gametophyte fully controls the energy supply level then *k* = 0 and *z_X_*_|_*_Y_* = *z_Y_*, whereas under full paternal control, *k* = 1 and *z_X_*_|_*_Y_* = *z_X_*.

In species in which the gametophyte is dioicous, the sexes are genetically determined, so males and females will have sex-determining regions—often a UV chromosome system. Our model assumes that the genes that determine the nutrient supply level to the sporophyte are not present on these sex chromosomes. In the moss *Ceratodon purpureus*, Shortlidge *et al*. [12] showed that a single female produced different numbers of spores when she was mated to two different males. This is clear support for male genetic variation, expressed in the sporophyte, for control over allocation from the female gametophyte to the sporophyte. Our assumption in the model (equation (2.7) with 0 < *k*) is consistent with this empirical fact.

Then, we constructed the next-generation matrix from the matrix on the right-hand side of (2.6) [25], and obtained the condition for the invasibility of a mutant allele. As described above, in our haploid–diploid system, the mutant allele can exist in a female mutant gametophyte (*F_a_*), in a female resident gametophyte with a mutant sporophyte $\left({\tilde{F}}_{A}\right)$, or in a male mutant gametophyte (*M_a_*). This ‘infection’ by a mutant allele can be evaluated in terms of the basic reproduction number used in epidemiology, *R*_0_, which can be calculated as the leading eigenvalue of the next-generation matrix. Using this method, we define the invasion fitness of a mutant with allelic effect *z_a_* for energy supply level from maternal gametophyte to a sporophyte in the population of residents with allelic effect *z_A_*:

2.8a $$\begin{array}{c} R_{0}=\frac{1}{2}\left[R^{Fa}+\sqrt{{\left(R^{Fa}\right)}^{2}+4mR^{FA}}\right], \end{array}$$

where

2.8b $$R^{FA}=\frac{1}{2m}\;\frac{\left(B(z_{a|A})/D((m-1)z_{A}+z_{a|A})\right)}{\left(B(z_{A})/D(mz_{A})\right)}$$

and

2.8c $$R^{Fa}=\frac{1}{2}\;\frac{\left(B(z_{A|a})/D(mz_{A|a})\right)}{\left(B(z_{A})/D(mz_{A})\right)},$$

where *z_a_*_|_*_A_* = *kz_a_* + (1 − *k*)*z_A_* and *z_A_*_|_*_a_* = *kz_A_* + (1 − *k*)*z_a_*. *R^FA^* is the expected number of *mutant* female gametophytes produced from a female ${\tilde{F}}_{A}$ that carries a heterozygote in one of her *m* sporophytes, relative to the expected number of female gametophytes produced by a female in the resident population. Similarly, *R^Fa^* is the expected number of mutant female gametophytes produced from a mutant female *F_a_* all of whose sporophytes are fertilized by resident males, relative to the expected number of female gametophytes produced by a female in the resident population. The condition for that the resident refuses invasion of the mutant, *R*_0_ < 1, is equivalent to that the following invasion fitness proxy *ϕ* is less than 1 (electronic supplementary material, appendix A):

2.9 $$\begin{array}{c} \phi =R^{Fa}+mR^{FA}<1.\; \end{array}$$

To check the model analysis, we numerically simulate a deterministic model and an individual-based model for a quantitative trait, the energy supply level *z*. Details are provided in electronic supplementary material, file S2.

## 3. Results

### (a) Evolutionarily stable energy supply

We first derive the condition under which the level of energy supplied from a haploid female to its diploid offspring is evolutionarily stable. By substituting (2.8*b*) and (2.8*c*) into (2.9), we see that the resident with allelic effect *z_A_* refuses invasion of a mutant with allelic effect *z* = *z_a_* if

3.1 $$\begin{array}{c} \frac{1}{2}\;\frac{B(z_{A|a})}{D(mz_{A|a})}+\frac{1}{2}\frac{B(z_{a|A})}{D((m-1)z_{A}+z_{a|A})}<\frac{B(z_{A})}{D(mz_{A})}. \end{array}$$

Conversely, if the left-hand side of (3.1) is larger than the right-hand side, then the mutant allele can invade the resident population. Hence, the arithmetic mean of the two types of expected reproductive success, *B*/*D*, gives the invasion fitness of a mutant when a genetic conflict exists. Here, *B*(*z_A_*_|_*_a_*)/*D*(*mz_A_*_|_*_a_*) represents the reproductive success of a mutant female gametophyte (whose mates are resident males; *F_a_*). By contrast, *B*(*z_a_*_|_*_A_*)/*D*((*m* − 1)*z_A_* + *z_a_*_|_*_A_*) represents the contribution to the invasion success of a mutant of a resident female gametophyte with one mutant sporophyte and *m* − 1 resident sporophytes (${\tilde{F}}_{A}$). This arithmetic mean of two terms can, therefore, be understood as the mean of male and female mutant invasion fitness (electronic supplementary material, appendix B).

#### (i) Optimization of reproductive success under maternal control

If the maternal allelic effect fully controls the energy supply, then *z_A_*_|_*_a_* = *z_a_*, *z_a_*_|_*_A_* = *z_A_* and *D*((*m* − 1)*z_A_* + *z_a_*_|_*_A_*) = *D*(*mz_A_*). Thus, (3.1) becomes

3.2 $$\begin{array}{c} \frac{B(z_{a})}{D(mz_{a})}<\frac{B(z_{A})}{D(mz_{A})}, \end{array}$$

and the resident allele *A* refuses the invasion of a mutant allele *a* controlling a similar trait, because the expected lifetime production of spores (*B*(*z*)/*D*(*mz*)) containing the resident allele is larger than that of spores containing the mutant allele. Conversely, a mutant allele can invade the resident population if the expected lifetime production of spores bearing the mutant allele from the female gametophyte is larger than that of spores bearing the resident allele. Therefore, the evolutionarily stable energy investment of the female gametophyte maximizes the expected lifetime production of spores, *B*(*z*)/*D*(*mz*).

#### (ii) Relation to the hypothesis of Searles

The above result for maternal control indicates that nonzero parental care in haploid–diploid plants can evolve when the selection gradient becomes positive at *z* = 0. This condition is equivalent to *B*′(0)*D*(0) − *mB*(0)*D*′(0) > 0, where *B*′(*x*) = d*B*(*x*)/d*x* and *D*′(*x*) = d*D*(*x*)/d*x* indicate the derivatives of the fertility and mortality functions. In particular, when the number of sporophytes is small (e.g. *m* ≈ 0) or the fertility of sporophytes without an energy supply is small (e.g. *B*(0) ≈ 0), it is easier for positive energy supply investment by the female gametophyte (parental care) to evolve. Because the number of sporophytes is proportional to the fertilization success rate (low fertilization success rates lead to small *m*), the facilitation of the evolution of such investment by a small number of sporophytes is consistent with the hypothesis of Searles [13].

We examine the evolutionarily stable investment for a given trade-off between the reproduction and mortality functions, *B*(*x*) and *D*(*x*). Let us assume here that the fertility of sporophytes and the mortality of female gametophytes can be expressed as (2.4). The energy supply level maximizes the expected total number of spores at

3.3 $$\begin{array}{c} \hat{z}=\frac{d_{0}\beta }{mc_{D}(1-\beta )} \end{array}.$$

In our model, the low fertilization success argued by Searles corresponds to a low *m*, the number of fertilized zygotes on a female gametophyte, and a low *m* causes the evolutionarily stable investment to be large, as can be seen from (3.3). In other words, when the level of investment is under maternal control, a female gametophyte tends to favour a higher investment in parental care when the fertilization success rate is low.

#### (iii) Unlimited exploitation under paternal control and multiple sporophytes

We next consider the case where the paternal, rather than the maternal, allelic effect completely determines the investment by the maternal haploid; that is, *z_A_*_|_*_a_* = *z_A_*, *z_a_*_|_*_A_* = *z_a_* and *D*((*m* − 1)*z_A_* + *z_a_*_|_*_A_*) = *D*((*m* − 1)*z_A_* + *z_a_*). Thus, (3.1) becomes

3.4 $$\begin{array}{c} \frac{B(z_{a})}{D((m-1)z_{A}+z_{a})}<\frac{B(z_{A})}{D(mz_{A})}. \end{array}$$

If there is only one sporophyte attached to the female gametophyte (*m* = 1), then *D*((*m* − 1)*z_A_* + *z_a_*) = *D*(*mz_a_*), and the evolutionary condition is the same as under maternal determination. However, when the number of sporophytes per maternal gametophyte (*m*) is very large, then *D*((*m* − 1)*z_A_* + *z_a_*) ≈ *D*(*mz_A_*) and the evolutionarily stable level of energy investment by female gametophytes is the maximization of the instantaneous rate of sporophyte reproduction, *B*(*z*), ignoring the mortality of maternal gametophytes, rather than the maximization of the lifetime production of spores, to which female longevity also contributes. We thus expect an unlimited evolutionary escalation of female investment in sporophytes at the expense of female longevity.

These two extreme cases (maternal versus paternal determination) suggest that, for more realistic intermediate degrees of maternal and paternal controls of investment in sporophytes, a sexual (genomic) conflict exists between selection for female genes, which because it considers the longevity of the maternal body (*B*(*z*)/*D*(*mz*)) favours a greater lifetime reproductive success of the female gametophyte, and selection for a selfish male gene, which favours a greater instantaneous rate of sporophyte reproduction (*B*(*z*)) without regard for the longevity of the female. In the next subsection, we examine the evolutionary outcome of this genomic conflict.

### (b) Evolutionary dynamics under genomic conflict

We here examine the evolutionary dynamics of the amount of energy supplied when both maternal and paternal allelic effects contribute to the amount of female investment in sporophytes, that is, 0 < *k* < 1 in (2.7). The selection gradient for the level *z* = *z_A_* of energy supplied by the female gametophyte to its sporophytes is

3.5 $$\begin{array}{c} S(z)=\frac{1}{2}\frac{B(z)}{D(mz)}\left(\frac{B^{\prime }(z)}{B(z)}-\alpha \frac{D^{\prime }(mz)}{D(mz)}\right), \end{array}$$

where *α* = *k* + *m*(1 − *k*) = (*m* − 1)(1 − *k*) + 1 > 0 is the effectiveness of maternal control of female gametophyte mortality, which is a function of the paternal allelic effect *k* and the number *m* of sporophytes. If the selection gradient is positive (S(z)>0) the energy supply level increases evolutionarily as a mutant that has slightly larger supply level than the resident can invade, while if S(z)<0 it decreases evolutionarily. An evolutionary equilibrium level of energy supply, called evolutionarily singular point, is defined as *z* = z* satisfying $S(z^{*})=0$, or

3.6a $$\begin{array}{c} G_{B}(z^{*})=\alpha G_{D}(mz^{*}), \end{array}$$

where

3.6b $$G_{B}(z)=\frac{B^{\prime }(z)}{B(z)}=\frac{d}{dz}\log B(z),$$

and

3.6c $$G_{D}(z)=\frac{D^{\prime }(mz)}{D(mz)}=\frac{1}{m}\frac{d}{dz}\log D(mz).$$

Noting that the sporophyte productivity *B*(*x*) is assumed to be an increasing and saturating function of the energy supply *x* from maternal gametophyte and that the maternal gametophyte mortality *D*(*x*) is assumed to be an increasing function of the energy *x* she supplies to her sporophytes, this implies that, for a fixed form of *D*(*x*), the slower the saturation of *B*(*x*), the higher the evolutionarily singular energy supply level z*. Focusing on the proportionality constant *α* in (3.6), we see that the stronger the maternal control (the larger is *α*) the smaller is the evolutionarily singular supply level z*.

The condition for the convergence stability of this evolutionary singular point (the condition under which the trait value evolves toward z* rather than evolutionarily diverges from z*) is that the derivative of the selection gradient at this evolutionarily singular point is negative (electronic supplementary material, appendix A), which is rewritten in terms of the logarithmic curvatures of sporophyte productivity *B*(*x*) and maternal gametophyte mortality *D*(*x*):

3.7a $$\begin{array}{c} \Gamma_{B}(z^{*})<\alpha m\Gamma_{D}(z^{*}), \end{array}$$

where

3.7b $$\Gamma_{B}(z)=\frac{B^{\prime \prime }(z)}{B(z)}-{\left(\frac{B^{\prime }(z)}{B(z)}\right)}^{2}=\frac{d^{2}}{dz^{2}}\log B(z),$$

and

3.7c $$\Gamma_{D}(z)=\frac{D^{\prime \prime }(mz)}{D(mz)}-{\left(\frac{D^{\prime }(mz)}{D(mz)}\right)}^{2}=\frac{1}{m^{2}}\frac{d^{2}}{dz^{2}}\log D(mz).$$

The evolutionarily singular energy supply level z* is convergence stable if sporophyte productivity saturates sufficiently more strongly with the energy supply level than the maternal gametophyte mortality does.

A convergence stable evolutionarily singular point can be evolutionarily unstable by allowing the invasion of nearby mutants of the resident. This leads to the evolutionary branching of the trait. The condition for evolutionary branching is that both (3.7) and the following inequality is satisfied (electronic supplementary material, appendix A):

3.8 $$\begin{array}{c} \frac{B^{\prime \prime }(x^{*})}{B(x^{*})}>\frac{k^{2}+{\left(\alpha -k\right)}^{2}}{k^{2}+{\left(1-k\right)}^{2}}\left(\frac{D^{\prime \prime }(mx^{*})}{D(mx^{*})}\right)+\frac{2k(1-\alpha )(\alpha -2k)}{k^{2}+{\left(1-k\right)}^{2}}G_{D}{\left(z^{*}\right)}^{2} \end{array},$$

where *G_D_*(*z*) is defined in (3.6*c*), and we used *G_B_*(z*) = *αG_D_*(z*) that holds at evolutionarily singular point. Conversely, the evolutionary singular point is evolutionarily stable when the inequality is reversed, that is, when the left-hand side of (3.8) is smaller than the right-hand side.

When the form of the mortality function is linear, we can drop the term proportional to *D*^′′^(mz*) = 0. In this case, evolutionary branching can occur when the following condition is satisfied:

3.9 $$\frac{2k(1-\alpha )(\alpha -2k)}{[k^{2}+{\left(1-k\right)}^{2}]\alpha^{2}}<\frac{B^{\prime \prime }(z^{*})}{B(z^{*})}/{\left(\frac{B^{\prime }(z^{*})}{B(z^{*})}\right)}^{2}<\frac{\alpha -m}{\alpha }.$$

The range of the scaled curvature of the fertility function at the evolutionary singular point z*, [*B*′′(z*)/*B*(z*)]/(*B*′(z*)/*B*(z*))^2^, where the supply level *z* shows evolutionary branching, is the area between the lower and upper bounds of (3.9) (shaded areas between the blue and red curves in electronic supplementary material, figure C1). From electronic supplementary material, figure C1, it is clear that (i) evolutionary branching can occur when the curvature of the scaled fertility function is appropriately negative and the allelic effect of the paternal gene *k* is smaller than a threshold value and (ii) the larger the number of sporophytes *m* per female gametophyte, the more easily evolutionary branching can occur. Inspection of the lower and upper bounds of (3.9) shows that evolutionary branching never occurs when the female has only a single sporophyte (*m* = 1) or when the energy supply level is maternally determined (*k* = 0). These results imply that the existence of sexual conflict is a prerequisite for evolutionary branching.

### (c) Effects of the degree of paternal determination and the number of sporophytes

We next use a pairwise invasibility plot to represent the evolutionary dynamics of the energy supply level *z*. To calculate the invasibility of mutant allele *a* against resident allele *A*, we use (2.4) as the function for the reproduction of sporophytes and the mortality of the female gametophyte. In this case, the evolutionary singular point can be described as

3.10 $$\begin{array}{c} z^{*}=\frac{d_{0}\beta }{c_{D}(\alpha -m\beta )}, \end{array}$$

where *α* = (*m* − 1)(1 − *k*) + 1. Note that the evolutionarily singular level of energy supply coincides with the optimum level $\hat{z}$ under maternal control case (*k* = 0) shown in (3.3). However, z* increases as *k* and *m* increase, and can be evolutionarily destabilized by sufficiently large *k* and *m*.

**Figure 3.**
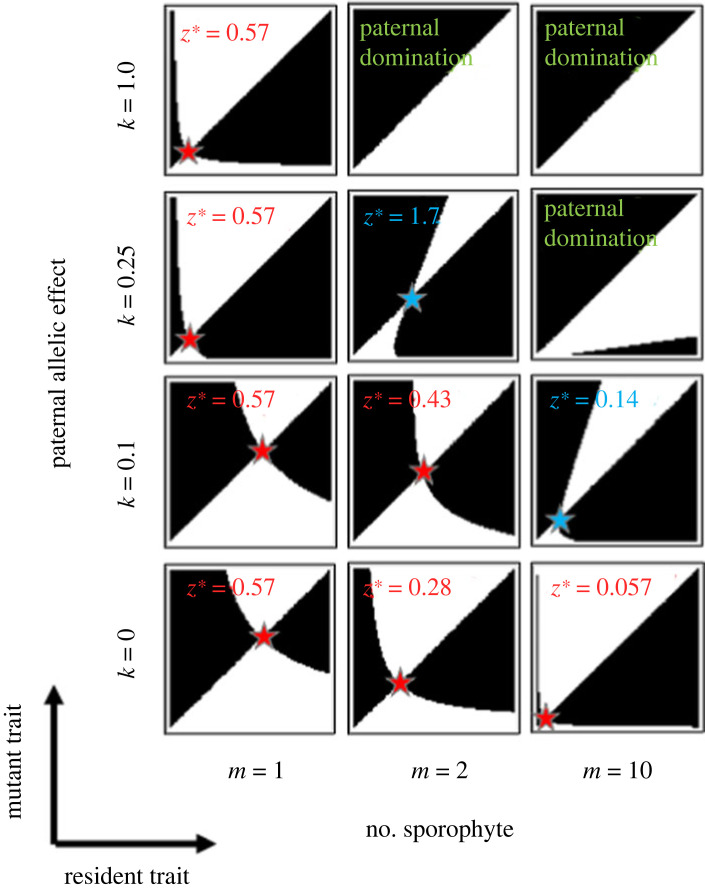
Pairwise invasibility plots for the evolution of energy investment, *z*, for different combinations of *m* (the number of sporophytes on the female gametophyte) and *k* (the allelic effect). We calculate the invasibility of mutant allele *a* against resident allele *A* using (2.6) as the function for sporophyte reproduction and gametophyte mortality. The *x* and *y* axes of each panel indicate the energy supply level of the resident (*z_A_*) and mutant (*z_a_*), respectively. Regions where the combination of resident and mutant traits allows the mutant allele to invade the resident population are coloured black. Regions where the trait combinations do not allow the mutant allele to invade the resident population are coloured white. Stars indicate evolutionary singular points: red stars indicate evolutionarily stable singular points (single ESS), and blue stars indicate evolutionarily unstable singular points (evolutionary branching). Other parameters are as follows: *c*_B_ = 100, *d*_0_ = 0.001, *c*_D_ = 0.01, *d*^M^ = 0.01, *β* = 0.85.

Figure 3 illustrates pairwise invasibility plots for different numbers of sporophytes on the female gametophyte (*m*) and different degrees of the paternal allelic effect (*k*). On these plots, the horizontal and vertical axes indicate the energy supply level of the resident (*z_A_*) and the mutant (*z_a_*), respectively. Black regions indicate combinations of resident and mutant traits that allow the mutant to invade the resident population, and white regions indicate trait combinations where the mutant allele cannot invade the resident population. The stars indicate evolutionarily stable (red) and unstable (blue) evolutionary singular points.

As represented by (3.2), the evolutionary singular points indicated in figure 3 maximize the expected lifetime reproductive success of the female gametophyte, *B*(*z*)/*D*(*mz*); these points are evolutionarily stable when the maternal gametophyte fully controls the energy supply level (*k* = 0). Similarly, there is a unique ESS (shown by a red star) when the number of sporophytes is one (effectively monogamy; *m* = 1), which maximizes the expected lifetime reproductive success of female gametophyte, *B*(*z*)/*D*(*mz*), for any *k*. Indeed, (3.1), the condition under which the resident allele refuses the invasion of the mutant allele when the resident and mutant traits are similar, becomes equivalent to (3.2), the condition under maternal control, when only one sporophyte grows on the female gametophyte (*m* = 1) whether the energy supply level is fully controlled by the maternal allele or the paternal allele (*k* = 0 or 1). These results show that sexual conflict does not arise when the reproductive system is effectively monogamous (*m* = 1). In strict terms, (3.6) shows that the evolutionary singular point satisfies the condition dln[*B*(*z*)]/d*z* = dln[*D*(*z*)]/d*z* for effective monogamy (*m* = 1, with which *α*(*k*, *m*) = 1 in (3.6)). Under this condition (ln[*B*(*z*)/*D*(*z*)])′ = 0, and the function *B*(*z*)/*D*(*z*) is maximized for any *k* at the evolutionary equilibrium.

**Figure 4.**
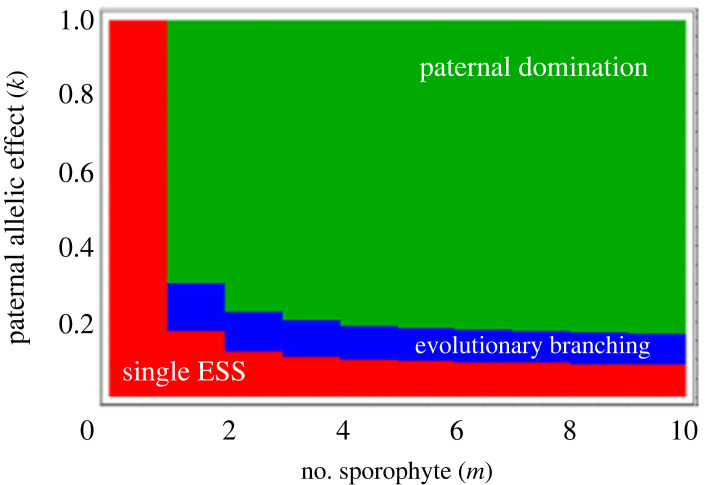
Regions in which each of the three evolutionary consequences arise for different values of *m*, the number of sporophytes on the female gametophyte, and *k*, the allelic effect. In the red region, there is a unique, evolutionarily stable energy supply level (single ESS); in the blue region, evolutionary branching occurs; and in the green region, the sporophyte receives as much energy as possible from the maternal body (paternal domination). Other parameters are the same as in figure 3.

However, as the number of sporophytes (*m*) and the paternal allelic effect (*k*) increase, the evolutionary outcomes dramatically change. When *m* ≥ 2, the energy supply level of an evolutionary singular point z* increases as the paternal allelic effect *k* increases (figure 3). Furthermore, this singular point becomes unstable (blue star) when *k* exceeds a certain threshold, leading to evolutionary branching. When *k* is further increased to pass a second threshold *k_c_*, defined below in (3.11), the singular point diverges to infinity, which means that female gametophytes evolve to increase their energy supply level without limit (or until the biological upper limit *z*_max_ is reached), and also means that the mortality of the female gametophyte is evolutionarily escalated without limit. We call this evolutionary result ‘paternal domination’. The parameter regions where the three different evolutionary consequences arise are shown in figure 4: (i) a unique, intermediate evolutionarily stable energy supply level (red region), (ii) evolutionary branching (blue region) and (iii) an energy supply to sporophytes from female gametophytes as large as possible (green region).

Under the trade-off (2.4), the evolutionarily singular investment z* in sporophytes is given by (3.12). By defining

3.11 $$\begin{array}{c} k_{c}=\frac{m(1-\beta )}{m-1}, \end{array}$$

which is a positive constant when the number *m* of sporophytes is greater than 1 (*m* > 1) and the trade-off is saturating (0 < *β* < 1), then

3.12 $$\begin{array}{c} z^{*}=\frac{d_{0}}{c_{D}}\frac{\beta }{m-1}\frac{1}{k_{c}-k}. \end{array}$$

The evolutionary singular supply level monotonically increases with the degree *k* of paternal control, and diverges to infinity as *k* approaches *k_c_*. The selection gradient (3.5) under the trade-off (2.4) becomes

3.13 $$\begin{array}{c} S(z)=\frac{1}{2}\frac{B(z)}{D{\left(mz\right)}^{2}}c_{D}(m-1)(k-k_{c})\left(\frac{z-z^{*}}{z}\right). \end{array}$$

In the region of strong paternal control (*k* > *k_c_*), z* becomes negative, and hence the selection gradient is positive for all supply level *z* > 0: in other words, indefinite evolutionary escalation of supply level occurs (paternal domination, in figures 3 and 4). By contrast, if the degree of paternal control is less than the threshold (*k* < *k_c_*), an intermediate evolutionarily singular investment strategy (3.12) exists, though the evolutionary stability of which depends on the curvature of trade-off (see below).

### (d) Evolutionary branching

As we see in figures 3 and 4, evolutionary branching can occur when *m* ≥ 2 and the value of *k* is intermediate. To observe the evolutionary dynamics of the polymorphic series after the evolutionary branching, we conduct numerical simulations with two models, a deterministic model and an individual-based model.

**Figure 5.**
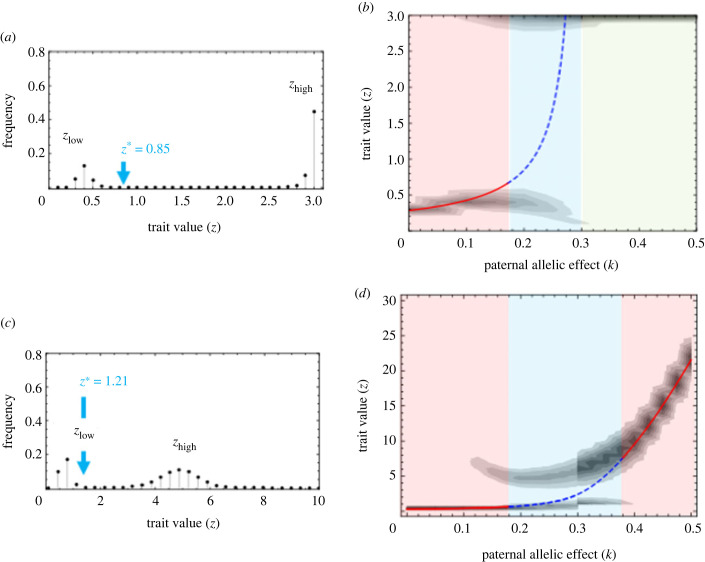
The evolutionary result of the deterministic model when there are two sporophytes on a maternal body (*m* = 2). (*a*,*c*) The frequency of trait values when paternal allelic effect has an intermediate value: (*a*) *k* = 0.2 and (*c*) *k* = 0.25. (*b*,*d*) Contour plot of the frequency of trait values for different values of the paternal allelic effect (*k*). (*a*,*b*) The result when we use *B*(*x*) = *c*_B_*x^β^* and (*c*,*d*) the result when we use *B*(*z*) = (*b*_max_*z^β^*)/(*c*_B_ + *z^β^*) for the fertility function. The solid red line indicates the values of the evolutionary singular point when it is evolutionarily stable. The blue dashed line indicates values of the evolutionary singular point when it is evolutionarily unstable. Three regions are delimited by the results of theoretical analysis: the area where there is a single ESS is red, that where evolutionary branching occurs is blue, and the region of paternal domination is green. Here, we set *b*_max_ = 100. The other parameters are the same as in figure 3.

The deterministic model with the parameter sets for evolutionary branching produces two peaks of *z* (figure 5*a*, where *k* = 0.2). Under the conditions that result in polymorphism, the population comprises two types of morphs, one with trait values around *z*_low_ and the other with trait values around *z*_high_. Figure 5*b* illustrates the evolutionarily stable energy supply levels for different values of the allelic effect *k*. When the maternal allelic effect is strong (red region), we tend to observe a single ESS that is equal in value to evolutionary singular point (red line). As the paternal effect (*k*) increases, the evolutionary singular point becomes unstable (blue dashed line) and evolutionary branching occurs (blue region). The mixture of these two types indicates that a female with *z*_low_ tends to be exploited by a sporophyte with the male gene with *z*_high_. Because the allele for *z*_high_ is maintained by this exploitation, its trait value evolves to become as high as possible until it finally reaches the preset maximum *z*_max_. When the paternal allelic effect dominates the evolution of the energy supply level (green region), paternal domination is observed. When *k* = 0.15, the evolutionary singular point is ‘locally’ evolutionarily stable (red region); however, a mutant that has a very large trait value can invade the population and genetic polymorphism is observed.

We note that this evolutionary result depends on the form of the fertility function *B*(*z*). If the fertility function cannot be infinitely large even with an infinitely large energy supply, then the degree of exploitation by the higher investment morph stops increasing at a finite level. Figure 5*c*,*d* shows the evolution of the dimorphism when we use a function of the form *B*(*z*) = (*b*_max_*z^β^*)/(*c*_B_ + *z^β^*), which saturates at large *z*.

**Figure 6.**
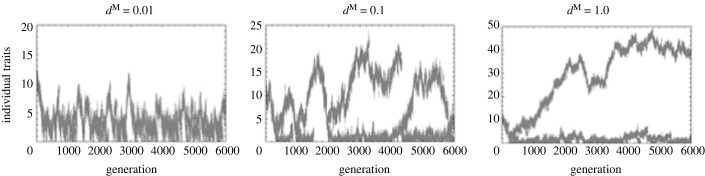
The results of the individual-based simulation for different values for the mortality rate of male gametophyte, *d*^M^, when *k* = 0.25. In this model, we simulate the population dynamics by the Moran model (see electronic supplementary material, C program file S2, for details). The total population size is fixed at 1000. Other parameters are the same as in figure 3.

Next, we examine the results of the individual-based simulation. Interestingly, this model result is strongly affected by the mortality rate of the male gametophyte, *d*^M^ (figure 6). With this model, we do not necessarily observe clear evolutionary branching within the parameter region where evolutionary branching occurs (low male mortality, *d*^M^ = 0.01). When the mortality of male gametophytes is increased, however, we observe evolutionary branching. When male mortality is intermediate (*d*^M^ = 0.1), the polymorphic state after evolutionary branching lasts only a short time, because a population with a morph with a low mean trait value goes extinct as a result of being over-exploited by a morph with a high mean trait value. After the extinction of the low-investment morph, the mean trait value of the now unimodal population decreases until it approaches the evolutionary branching point and the evolutionary branching occurs again. When the mortality of the male gametophyte is high (*d*^M^ = 1.0), the polymorphic state is stably maintained.

## 4. Discussion

While the evolution of parental care in animals is one of the hottest research topics in classical behavioural ecology, parental care in plants (and algae) has long been focused exclusively on seed plants. In this paper, we examined this topic by analysing the evolution of energy investment by a haploid parent (i.e. the female gametophyte) to its diploid offspring (i.e. sporophytes in bryophytes, and carposporophytes in red algae).

Motivated by two verbally expressed hypotheses [10,13], we have proposed a simple population genetic model with a haploid–diploid life cycle and examined the evolutionarily stable level of maternal investment (parental care) (*z*) in diploid sporophytes (offspring) by a haploid female gametophyte (mother). We found that the number of sporophytes (or zygotes) on a female gametophyte (*m*) and the degree of paternal versus maternal control (*k*) of the investment level are critical to the evolutionary outcome.

### (a) Sexual (genomic) conflict between female and male genetic effects

We first examined the evolution of parental care when a haploid female fully controls the investment. Under maternal control, the expected lifetime reproductive success of female gametophytes should be maximized through evolution. In particular, when the number of sporophytes (or fertilized eggs) attached to a female gametophyte is small, it is easier for a positive energy supply investment by the female gametophyte (parental care) to evolve, and the evolutionarily stable investment can be large.

Classically, parental care in haploid–diploids (i.e. triphasic life cycle in red algae) has been explained by the hypothesis of Searles [13]. Under this hypothesis, parental care is explained as an adaptive strategy of maternal gametophytes to compensate for a low fertilization success rate. In our model, the low fertilization success argued by Searles [13] corresponds to a small number of sporophytes attached to the maternal gametophyte, and under such conditions our model predicts a higher investment in parental care under maternal determination. Thus, our model provides theoretical support for his hypothesis. However, female gametophytes of red algae generally have many carposporophytes and some empirical studies [19–21] suggest they could have high fertilization success rates. Then the evolution of parental care in red algae should be discussed by different mechanisms, e.g. the conflict between males and females, as we explored in the present paper.

The genomic conflict between maternal and paternal alleles complicates the evolutionary forces acting on maternal investment in sporophytes. Selection acting on maternal genes maximizes the lifetime reproduction of the maternal gametophyte (*B*/*D*) by adjusting the energy investment. However, the allele from the male gametophyte in the sporophyte tends to favour a higher maternal energy investment even if that investment decreases the longevity of the maternal gametophyte. In extreme cases, when the male allelic effect is completely dominant (*k* = 1) and the number of sporophytes is very large (*m* ≫ 1), the selection pressure maximizes the instantaneous reproduction of sporophyte (*B*) and the maternal gametophyte supplies as much energy as possible until the physiological limit (*z*_max_) is reached.

This result can be understood as selfishness of the paternal allele in the diploid sporophyte. Because only the female gametophytes bear the cost of parental care (energy supply), the paternal genes in sporophytes try to obtain as high an energy supply as possible from the female gametophyte, thus accelerating the evolution of parental care. In particular, because sporophytes attached to the same maternal gametophyte do not have the same paternal gene (i.e. sporophytes on a female gametophyte are half-sibs), there is no motivation to mitigate the selfish behaviour for kin; thus, the sporophytes compete for the ‘commons’ (i.e. the resources provided by the maternal gametophyte). As previous researchers have suggested by verbal argument [10,23,24], genetic conflict can be an important factor in the evolution of traits relating to parental care in plants. Interestingly, even when there is only one sporophyte (*m* = 1), the paternal gene favours the greater lifetime reproductive success of the maternal gametophyte, so no genomic conflict arises in that case.

The number of sporophytes on a female gametophyte is much larger in red algae (e.g. *m* > 100) than in mosses, pteridophytes, and brown algae, which tend to have fewer sporophytes (e.g. *m* = 1). Therefore, the hypothesis of Searles [13] can more plausibly account for the evolution of parental care in them, whereas the hypothesis of Haig & Wilczek [10] is more plausible in red algae. We note that if perennial female gametophytes nurture several sporophytes in succession, however, because of the mate changes, one would expect the selfishness of the paternal genes to be stronger and energy exploitation would be observed even in the case of *m* = 1. This means that the view of Haig & Wilczek [10] becomes important even for bryophytes (see Model extensibility for the diverse life cycles).

In empirical studies, there are several studies that follow Searles' hypothesis. For example, Kamiya & Kawai [18] were pioneering in their indirect study of nutrient supply from gametophyte to sporophyte. There are also studies that question the premise of Searles’ hypothesis by examining fertilization success rates in red algae [19–21]. However, studies based on the genomic conflict hypothesis are still scarce (but see [12]). Our study suggests the importance of genomic conflict in the evolution of parental care, and future empirical studies in a wide range of taxa (i.e. other than red algae; bryophytes, pteridophytes and brown algae) are required.

### (b) Evolution of parental care under genomic conflict

In the haploid–diploid system considered here, there are three possible outcomes of the evolution of parental investment. (i) When the number of sporophytes *m* is small or the maternal allelic effect is strong (small *k*), then evolutionary equilibrium, an evolutionary singular point, is reached at an intermediate investment level (i.e. a level that is both evolutionarily and convergence stable, or continuously stable)—the mean trait evolves toward this level, and once it is reached, the resident allele refuses the invasion of any other mutants (red region in figure 4). The evolutionary equilibrium investment level increases as the selfishness of the parental gene becomes more influential, either by an increase in the number of sporophytes (*m*) or by an increase in the paternal allelic effect (*k*). (ii) By contrast, if the parameters enhancing the temptation for paternal selfishness (i.e. *m* and *k*) become too large such that they exceed a threshold, the intermediate evolutionary equilibrium investment level disappears, and the female's investment increases evolutionarily without limit or until it hits a physiological upper limit, resulting in an excessive level of parental care (green region in figure 4). (iii) When the male and female genes contribute similarly to the determination of the investment level, then the intermediate singular point is no longer evolutionarily stable; evolutionary branching occurs, and two morphs are produced, one with a high- and the other with a low-investment genotype (blue region in figure 4). In outcomes (ii) and (iii), the female gametophyte suffers from a mortality risk caused by excessive investment in sporophytes driven by paternal selfishness.

Interestingly, a structure specific to red macroalgae is a structure called a ‘fusion cell’. When syngamy occurs in red macroalgae, a diploid cell with maternal and paternal genes fuses with a structure of the maternal gametophyte called an auxiliary cell, giving rise to a polyploid fusion cell. This auxiliary cell is characterized by diversity among species [16, pp. 422–425], but has been insufficiently considered from an evolutionary perspective. Because a carposporophyte grows from this fusion cell, the fusion cell may control the level of investment in the diploid carposporophyte. If so, then the development of fusion cells may act as an evolutionary counterforce that allows the maternal gametophyte to suppress the selfishness of the paternal allele (lowering the value of *k* in our model). Similar logic may explain clonal reproduction and selective abortion in macroalgae. Lavaut *et al*. [26] pointed out the possibility of the female choice after fertilization in red algae as a context of sexual selection. If female gametophytes can use their energy for clonal growth other than parental care, or selectively abort the sporophytes containing paternal gene with higher selfishness, these could indirectly decrease the disadvantage caused by genomic conflicts (see next subsection). Thus, our model can help us understand the evolution and diversity of various life cycle strategies in haploid–diploids.

If a single ESS is achieved through the amplification maternal allelic effects, then the mortality of the female gametophyte is determined by the total energy supply mz*. Electronic supplementary material, figure C2, illustrates the parameter dependence of mz* (see (3.10)); the total amount of nutrients supplied by the female gametophyte increases as the number of sporophytes *m* increases. Thus, the greater the number of sporophytes, the higher the mortality of the female gametes caused by the selfishness of the paternal allele. In the case of red macroalgae, this trend should also be related to the size of the fusion cell. It would be interesting to investigate whether correlations among the number of sporophytes, the size of the fusion cell, and the mortality of female gametophyte can be observed in nature.

Evolutionary branching is difficult to interpret biologically, but if this phenomenon occurs in the field, one would expect to observe genetic polymorphisms in which individuals with different energy investment levels coexist in a population. In fact, Shortlidge *et al*. [12] revealed that genotypic variation exists in field populations and that their combination results in widely varying spore reproduction. Our model may partially explain this maintenance of genetic variation in the field.

### (c) Model extensibility for the diverse life cycles

In our model, target species are assumed to be annual, dioicous, and obligate sexuality. However, this assumption often does not hold. Therefore, we discuss here what can arise when these assumptions are relaxed.

First, in our model, when the number of offspring is one (*m* = 1), there is no genomic competition. However, this result depends on the assumption of no change in mate. For example, if the species is perennial and reproduces over many years, or exhibits regeneration from the basal part, the mate (the paternal allele in sporophytes) changes with each regeneration. In this case, the interests of the maternal and paternal genes no longer coincide. We would expect the selfishness of the paternal genes to be stronger and energy exploitation would be observed even in the case of *m* = 1.

Second, we consider the case when the species exhibits hermaphrodites. In this case, the evolutionary results would strongly depend on the degree of outcrossing. For example, if 100% outcrossing occurs, the conclusion should be the same as in the case of dioicous because the paternal gene in sporophyte is not related to maternal gene as is in the case of dioicous. By contrast, if the species exhibits 100% selfing, there is no genomic conflict because the interests of the paternal and maternal genes in sporophyte coincide. Hence, an intermediate degree of outbreeding would weaken genomic conflicts.

Third, gametophytes often reproduce by asexual reproduction (e.g. many red algae have multiple ramets). If asexual reproduction occurs before sexual reproduction (development of sporophytes), this might not affect the evolution of parental care, because this is just a ‘growth’ of the gametophytes. If the asexual reproduction occurs after the sexual reproduction, however, female gametophytes can escape from the exploitation of paternal genes by investing the available energy into the asexual reproduction. This option would probably weaken genomic conflicts by favouring maternal genes.

### (d) Future perspectives

Although we succeeded in mathematically describing the main hypotheses and obtaining clear evolutionary outcomes, our model has some potential limitations and unsolved problems that should be addressed in the future. First, the relationship between the evolution of parental care and population dynamics is still insufficiently understood. In our model, we assume that the total population size remains constant. Because of this assumption, even when the maternal gametophyte supplies a huge amount of energy to sporophytes under paternal gene dominance, causing the longevity of the female gametophyte to become very short, the population remains stable. However, if this simplifying assumption is relaxed, then we might expect that a population dominated by the paternal effect male gene would cease the evolutionary escalation caused by the selfishness of the male gene before the population is driven to extinction (though evolutionary suicide would not be ruled out). Furthermore, we assume that sperm limitation does not occur. If the frequency of male gametophytes in the population becomes very low, we could also model a decrease in the sporophyte number *m* due to the occurrence of sperm limitation.

Second, a similar sexual conflict is known from the evolution of imprinting in mammals [27]. In the maternal body, the paternal gene often represents selfishness, causing a genomic conflict for the energy supply to arise. Although in our model we assumed that the inheritance of the level of investment was governed by a single locus, Mochizuki *et al*. [27] assumed that maternal and paternal traits were coded by independently imprinted genes and that the paternal gene could easily dominate and drive the evolution of the investment level. It would be interesting to ask in future work how our results would be changed if we also assumed the existence of two loci coding maternal and paternal effects in our model.

Until recently, studies on genomic conflicts in plants have been limited to the evolution of endosperm (double fertilization) in seed plants [6]. We highlighted nutrient supply from a haploid female gametophyte to its diploid offspring, which is widely observed in bryophytes, pteridophytes and red algae, as a new area in which genomic conflicts are essential, and presented testable hypotheses for the evolution of the fusion cells in red algae that occur there. Future applications of mathematical modelling to the taxa other than seed plants, such as those presented in this paper, are expected to advance our understanding of issues fundamental to plant ecology.

## Data Availability

The details of mathematical analysis and program files are openly available as electronic supplementary material. One can regenerate original datasets for figures using these program files [28].
